# CCL2 Serum Levels and Adiposity Are Associated with the Polymorphic Phenotypes -2518A on CCL2 and 64ILE on CCR2 in a Mexican Population with Insulin Resistance

**DOI:** 10.1155/2016/5675739

**Published:** 2015-12-29

**Authors:** Milton-Omar Guzmán-Ornelas, Marcelo Heron Petri, Mónica Vázquez-Del Mercado, Efraín Chavarría-Ávila, Fernanda-Isadora Corona-Meraz, Sandra-Luz Ruíz-Quezada, Perla-Monserrat Madrigal-Ruíz, Jorge Castro-Albarrán, Flavio Sandoval-García, Rosa-Elena Navarro-Hernández

**Affiliations:** ^1^Instituto de Investigación en Reumatología y del Sistema Musculo Esquelético, Centro Universitario de Ciencias de la Salud, Universidad de Guadalajara, Sierra Mojada No. 950, Colonia Independencia, 44340 Guadalajara, JAL, Mexico; ^2^UDG-CA-701, Grupo de Investigación Inmunometabolismo en Enfermedades Emergentes (GIIEE), Centro Universitario de Ciencias de la Salud, Universidad de Guadalajara, Sierra Mojada No. 950, Colonia Independencia, 44340 Guadalajara, JAL, Mexico; ^3^Translational Cardiology, Center for Molecular Medicine, Department of Medicine, Karolinska Institutet, L8:03, 17176 Stockholm, Sweden; ^4^Departamento de Biología Molecular y Genómica, Centro Universitario de Ciencias de la Salud, Universidad de Guadalajara, Sierra Mojada No. 950, Colonia Independencia, 44340 Guadalajara, JAL, Mexico; ^5^Servicio de Reumatología, División de Medicina Interna, Hospital Civil “Dr. Juan I. Menchaca”, Universidad de Guadalajara, Salvador de Quevedo y Zubieta No. 750, 44340 Guadalajara, JAL, Mexico; ^6^Departamento de Disciplinas Filosófico, Metodológico e Instrumentales, Centro Universitario de Ciencias de la Salud, Universidad de Guadalajara, Sierra Mojada No. 950, Colonia Independencia, 44340 Guadalajara, JAL, Mexico; ^7^Departamento de Farmacobiología, Centro Universitario de Ciencias Exactas e Ingenierías, Universidad de Guadalajara, Boulevard Marcelino García Barragán No. 1421, 44430 Guadalajara, JAL, Mexico

## Abstract

Genetic susceptibility has been described in insulin resistance (IR). Chemokine (C-C motif) ligand-2 (CCL2) is overexpressed in white adipose tissue and is the ligand of C-C motif receptor-2 (CCR2). The *CCL2* G-2518A polymorphism is known to regulate gene expression, whereas the physiological effects of the *CCR2*Val64Ile polymorphism are unknown. The aim of the study is to investigate the relationship between these polymorphisms with soluble CCL2 levels (sCCL2), metabolic markers, and adiposity. In a cross-sectional study we included 380 Mexican-Mestizo individuals, classified with IR according to Stern criteria. Polymorphism was identified using PCR-RFLP/sequence-specific primers. Anthropometrics and metabolic markers were measured by routine methods and adipokines and sCCL2 by ELISA. The CCL2 polymorphism was associated with IR (polymorphic *A+* phenotype frequencies were 70.9%, 82.6%, in individuals with and without IR, resp.). Phenotype carriers CCL2 (*A+*) displayed lower body mass and fat indexes, insulin and HOMA-IR, and higher adiponectin levels. Individuals with IR presented higher sCCL2 compared to individuals without IR and was associated with CCR2 (*Ile+*) phenotype. The double-polymorphic phenotype carriers (*A+/Ile+*) exhibited higher sCCL2 than double-wild-type phenotype carriers (*A−/Ile−*). The present findings suggest that sCCL2 production possibly will be associated with the adiposity and polymorphic phenotypes of *CCL2* and *CCR2*, in Mexican-Mestizos with IR.

## 1. Introduction

The insulin resistance (IR) presents many subclinical manifestations, characterized by alterations in lipids and carbohydrates metabolism at different levels. Most of these changes is due to a low-grade systemic chronic inflammation [1, 2]. Adipose tissue is the primary anatomical site where the disease takes place. In early stage, this tissue became inflamed with the following pathological mechanism: first, monocytes migrate to adipose tissue, these cells express high levels of C-C motif receptor 2 (CCR2) and release monocyte chemoattractant protein-1 (MCP-1), also known as chemokine (C-C motif) ligand 2 (CCL2). This chemokine can promote further local inflammation and/or acts in paracrine way. The signaling through CCR2 may polarize the monocytes to M1 macrophages; these cells present a proinflammatory profile. Nevertheless, CCL2-CCR2 interaction is known to regulate continuous migration of monocytes to adipose tissue [3–5].

CCL2 is produced in soluble form by monocytes and macrophages and binds with high affinity to the CCR2 receptor. The later one is constitutively expressed in monocytes and its levels decrease as it differentiate into macrophages [4].

The human* CCL2* gene is located on chromosome 17q11.2 [6, 7]. It has two remote kappa B binding sites known as A1 (-2640/-2632) and A2 (−2612/−2603) that regulates the transcription of* CCL2* gene. Whereas the* CCR2* belongs to the family of seven transmembrane-spanning receptors that are coupled to heterotrimeric G proteins, the gene is located on the chromosome 3p21 within a cluster of chemokine receptor genes [7, 8].

The IR is considered a multifactor and polygenic disease; nevertheless, it has not determine the environmental and genetic factors contribution. In this context the study of candidate genes to make a contribution in clarifying this point is important. It has been reported that single nucleotide polymorphisms (SNP) in* CCL2* and* CCR2* are related with IR, G-2518A (rs 1024611), and Val64Ile (rs 17998649), respectively. The SNP of* CCL2* is located at 85 base pairs (bp) of remote kappa B binding site A2, while in* CCR2* gene, the SNP is conservative and the amino acid change (Val>Ile) takes place in the first transmembrane domain. This change decreases the affinity CCL2 binding, since the join of CCL2 with CCR2 receptor is through the second transmembrane domain [9–11].

Clinical phenotype has been described with these polymorphisms; type 2 diabetes mellitus (T2DM), cardiometabolic risk factors, obesity indexes, and insulin secretion association were found [12]. Interestingly, another study failed to show effect of this polymorphism in adipokines levels in patients with essential hypertension and T2DM [13]. Studies in animal models were performed to demonstrate the functional effect of these polymorphisms, but the results were not conclusive [14].

With this in mind, the aim of this study is to describe the presence of polymorphism of* CCL2* and* CCR2* in a population with IR, compared to healthy subjects, and to elucidate the clinical/metabolic features that these polymorphisms may present.

## 2. Material and Methods

### 2.1. Subjects' Assessment

In this cross-sectional study, a total of 380 nonrelated Mexican-Mestizos (i.e., an individual that were born in Mexico, with a Spanish last name and a family history of Mexican ancestors for at least three generations), and aged 20–69 years, were recruited from population of Western Mexico and classified according to Stern criteria in two groups: group 1 individuals with IR, if any of the following conditions were met: HOMA-IR > 4.65 or BMI > 27.5 kg/m^2^ and HOMA-IR > 3.6, and group 2 individuals without IR, therefore negative for those who did not meet the above conditions (i.e., HOMA-IR ≤ 4.65 or BMI ≤ 27.5 kg/m^2^ and HOMA-IR ≤ 3.60) [15]. Inclusion criteria for the study were considered as follows: individuals who at the time of the study did not present glucose intolerance, infectious diseases, hypertension, history of cardiovascular disease, malignancy, and renal and metabolic diseases such as T2DM. The subjects were questioned and denied any medication or weight change at least 3 weeks.

### 2.2. Ethics Conduct

Before enrolment, participants were informed about the study and signed a consent form following the Helsinki declaration guidelines, and the institutional (Guadalajara University) review boards' committees ensured appropriate ethical and biosecurity conduct [16].

### 2.3. Medical History and Physical Examination

All individuals who fulfil inclusion criteria were clinically evaluated by a physician who performed a complete medical history and assessment of general health status and vital signs were included: blood pressure (executed 3 times with the subject in the sitting position and relaxing for 15 minutes before the measurement), heart and respiratory rate, and body temperature.

### 2.4. Body Fat Storage Measurements

We evaluated the following body measurements: height, measured to the nearest 1 mm by using a stadiometer (Seca GmbH & Co. KG. Hamburg, Germany), weight, body mass index (BMI), and total body fat, determined by using bioelectrical impedance analysis (TANITA TBF304.Tokio, JPN) to the nearest 0.1 kg. Waist and hip circumferences where measured to the nearest 0.1 cm by using an anthropometric fiberglass tape (GULICK length 0–180 cm precision ±1 mm; USA) following the procedures recommended by the anthropometric indicators measurement guide [17, 18]. We calculated the waist-hip ratio [19] [WHR = waist (cm)/hip (cm)], body fat ratio [BFR = body fat mass (kg)/height^2^ (m^2^)], and waist to height ratio [WHtR = waist (cm)/height (cm)], as indicators of adiposity [20, 21].

### 2.5. Laboratory Techniques and Procedures

Individuals included in the study were fasting 12 hours before the blood samples were taken, after allowing them to clot at room temperature; then the blood was centrifuged at 1509 RCF (Rotanta 460R, Andreas Hettich GmbH & Co. KG) for 10 minutes at 20°C. Serum was collected and stored at −86°C until further analysis. We quantified the serum concentration of C reactive protein (CRP, with a limit of detection of 0.15 mg/L), basal glucose, lipid profile that included triglycerides, total cholesterol, HDLc, LDLc, and VLDLc (high, low, and very low density lipoprotein cholesterol, resp.), and apolipoproteins A1 and B (Apo-A1 and Apo-B, Randox Laboratories 55 Diamond Road, Crumlin Co. Antrim, Northern Ireland UK). By using commercial enzyme-linked immunoabsorbent assays (ELISA) were determined soluble levels of insulin (sensitivity of 0.399 *μ*UI/mL), sCCL2 (limit of detection of 2.3 pg/mL), sAdiponectin (limit of detection 0.019 ng/mL) (ALPCO Diagnostics 26-G Keewaydin Drive, Salem, NH), and sResistin (sensitivity 0.026 ng/mL, R&D Systems Inc., Minneapolis, MN, USA).

### 2.6. SNPs Analysis

Genomic DNA was obtained from total blood using a standard protocol for extraction with the modified Miller method as described previously [22] and was stored at −20°C until being used for genotyping. For each gene studied, polymorphic regions were amplified by polymerase chain reaction (PCR) method as described previously [23, 24].

To analyze the* CCR2* Val64Ile SNP was determined using sequence-specific primers (SSP): forward, Val 5′-TGGGCAACATGCTGGTCG-3′, Ile 5′-TGGGCAACATGCTGGTCA-3′, and reverse: 5′-TGGAAAATAAGGGCCACAGAC-3′ annealing 62°C, PCR product 413 bp [11], and* CCL2* G-2518A SNP, forward primer 5′-TCACGCCAGCACTGACCTCC-3′, and reverse: 5′-ACTTCCCAGCTTTGCTGGCTGAG-3′ with annealing temperature 56°C, PCR product 250 bp.

The PCR were performed in a 25 *μ*L total volume (mixture with 100 ng of DNA, 2 nM of each primer, 0.20 mM of each dNTP, 0.25 U* Taq* polymerase, and 1x PCR buffer) and 1.5 or 3.0 mM of MgCl_2_ for* CCL2* or* CCR2*, respectively.

To determine* CCL2* genotypes a PCR was performed and then a digestion of obtained products with* PVU II* restriction enzyme. The lengths of fragments observed were as follows: 175 and 75 bp (allele A) and 250 bp (allele G). Electrophoresis was done at a constant voltage of 180 V on 6% polyacrylamide gels stained with silver nitrate. For quality control, a blank and samples previously confirmed as positive for each genotype were used as controls. To ensure the accuracy of genotype data, we used internal controls and repetitive experiments. In addition, both polymorphisms were identified in duplicate by two different analysts. The genotyping success rate was 100%.

### 2.7. Statistical Analysis

Data were analyzed with the Statistics program SPSS v21 (IBM Inc., Chicago, IL, USA) and GraphPad Prism v6.01 (2014 Inc. 2236 Beach Avenue Jolla, CA 92037). Results are given as mean ± SD or median with 25, 75 percentiles or percentages based on normal distribution. The data distribution of clinical and laboratory variables of the study group was evaluated with *Z* Kolmogorov-Smirnov test, and we performed parametric and nonparametric test, as appropriate. The most important variables were adjusted by gender and age with an ANCOVA analysis. About these results we performed multifactorial analysis for the most important variables. The clinical and laboratory characteristics of the study group were performed with the unpaired Student's *t*-test or Mann-Whitney *U* test, and to compare quantitative data in four groups, a one-way ANOVA and post hoc Tukey test were used.

Data from serum concentrations of adipokines, the laboratorial assessment, and disease variables were subjected to Pearson or Spearman correlation tests. The Hardy-Weinberg equilibrium text for individual* loci* was performed with http://ihg.gsf.de/cgi-bin/hw/hwa1.pl. Contingency tables (2 × 2 and 2 × 3) with *χ* ^2^ trend test or Fisher exact test, as appropriate, were used for testing the differences of genotype distribution and allele frequencies between study groups. Two genetic models were used for these analyses: (1) the dominant model where each SNP was modeled categorically and separated into three categories, one for each genotype, and (2) the phenotype model, where each SNP was modeled into two categories, with two genotypes combined into one category (polymorphic homozygotes plus heterozygotes), choosing one genotype (homozygotes wild type) as the reference group. A two-tailed *P* value less than 0.05 was considered statistically significant.

## 3. Results

### 3.1. Adiposity Is Associated with Metabolic Markers

Anthropometrics characteristics and metabolic markers of the subjects included in this study split by IR Stern classification are shown in Table 1. The study group included 380 Mexican-Mestizo individuals of which 237 (62%) were women, they were classified without IR or with IR, ANCOVA was performed, adjusted for sex and age, and no differences were observed (data not showed). Twenty-one percent has been classified with obesity and 32% with IR. Sixty-five percent of individuals, included in the study, were determined with excess body fat according to the Deurenberg criteria [21], and 31% had dyslipidemic profile (data no shown). A positive correlation of soluble levels of sCCL2, adipokines, metabolic markers, and lipid profile (except HDLc, LDLc, and Apo-A1) was observed along body fat storage (Table 2).

### 3.2. Individuals with IR Presented Inflammatory State

**Figure 1:**
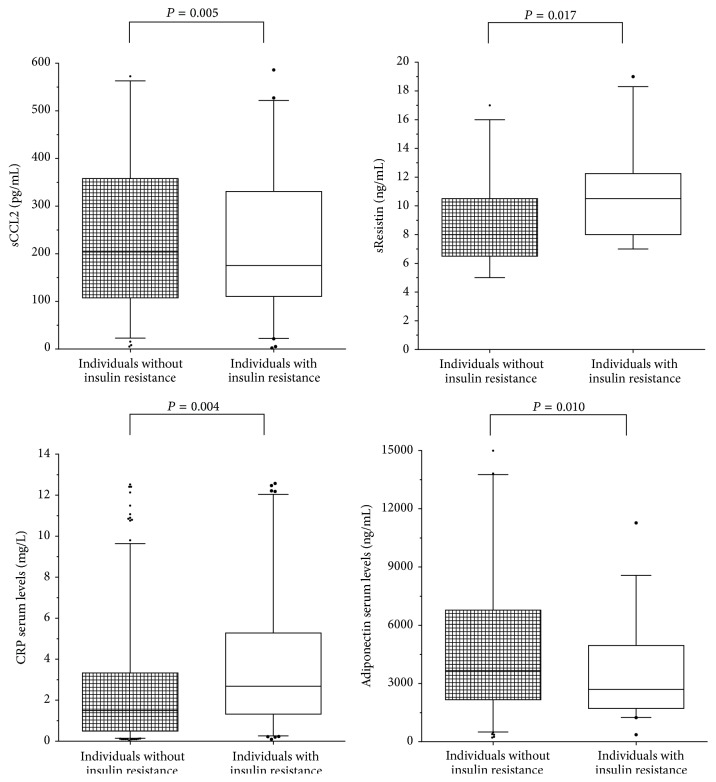
Soluble levels of adipokines and CRP by study group. *n* = 380. Student's  *t*-test with *P* significantly, comparing the groups: individuals with IR versus individuals without IR.

According to IR classification, individuals with IR displayed higher soluble levels of CCL2, resistin, and CRP and lower levels of adiponectin than individuals without IR (Figure 1). Soluble levels of CCL2, CRP, sResistin, and metabolic markers correlated positively with body adiposity, whereas levels of soluble adiponectin correlated negatively (Table 2).

### 3.3. CCL2 Polymorphism Is Associated with IR

**Figure 2:**
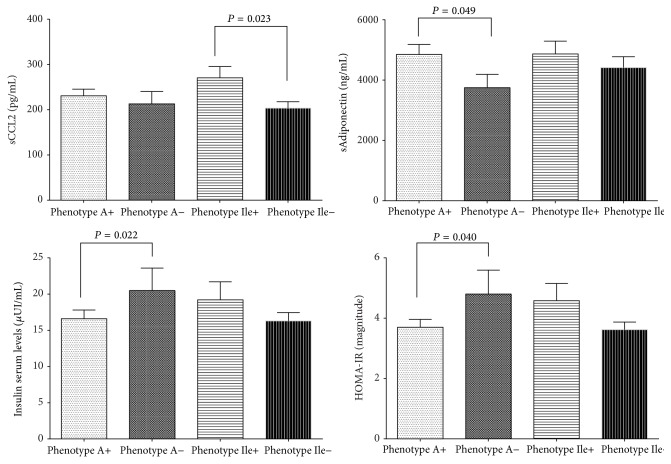
Soluble levels of adipokines, insulin, and HOMA-IR by* CCL2* G-2518A and* CCR2* Val64Ile phenotype carriers. *n* = 380. A+ phenotype: A/A plus G/A genotypes; Ile+ phenotype: Ile/Ile plus Val/Ile genotypes. Student's  *t*-test with *P* significantly, comparing the polymorphic phenotype carriers versus wild-type phenotypes carriers.

All genotype frequencies were in Hardy-Weinberg equilibrium. The individuals without IR presented a higher frequency of the phenotype* A+* of the* CCL2* polymorphism, while phenotype* A−* is more common in the individuals with IR. These differences were also observed in genotype and allele frequencies (Table 3). Higher levels of total adiponectin as well as a parallel decrease in insulin levels and HOMA-IR index were associated with* A+* phenotype carriers (Figure 2), as long as phenotype* A+* was associated with low measures of BMI and BFR (Table 4).

### 3.4. CCR2 Polymorphism Was Not Associated with IR but Promoted Clinical Features of IR

None of the* CCR2* variants had an association with IR (Table 3). The detailed analysis showed association of phenotype* Ile+* with high BMI, levels of glucose and lipids (Table 4). The same as* CCL2 A+* phenotype, in* CCR2 Ile+* carriers presented higher levels of CCL2 compared to* Ile−* phenotypes (Figure 2).

### 3.5. A+ Phenotype of CCL2 and Ile+ of CCR2 Are Associated with High Levels of Circulating CCL2

**Figure 3:**
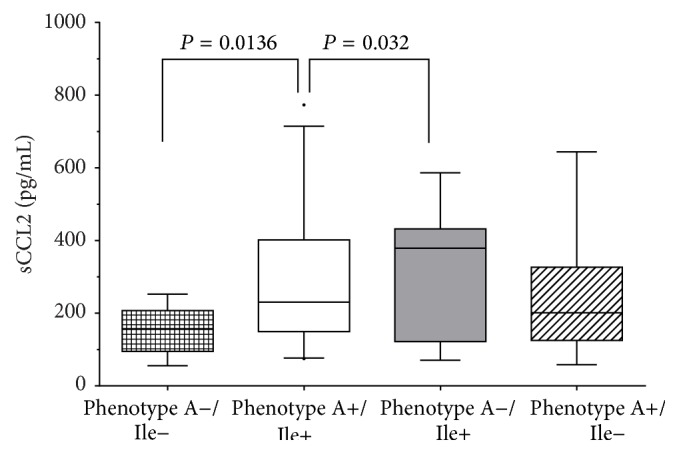
Soluble levels of CCL2 by phenotypes. Phenotype A−/Ile− (genotypes GG/ValVal) *n* = 48; phenotype A+/Ile+ (genotypes AA plus GA/ValIle plus IleIle) *n* = 108; phenotype A−/Ile+ (genotypes GG/ValIle plus IleIle) *n* = 32; phenotype A+/Ile− (genotypes AA plus GA/ValVal) *n* = 192. One way ANOVA and Tukey's post hoc test with *P* significantly, comparing the double-polymorphic phenotype carriers, (A+/Ile+) versus double-wild-type phenotype carriers (A−/Ile−).

No metabolic profile explored in this study was associated with A+ phenotype (Table 4). However, significant phenotype-by-phenotype association was observed between the* CCL2*-2518A allele and the* CCR2* 64Ile allele; carriers of the* CCR2* phenotype 64Ile+ who at the same time also were phenotype A+ of* CCL2* (polymorphic genotypes for the both polymorphisms) presented higher levels of soluble CCL2, than any other phenotype combinations (Figure 3).

## 4. Discussion

The IR is a disease of multifactorial etiology product of the interaction between the genetic component and the environment, epidemiological data describes IR as an emerging disease with epidemic proportions [19, 25, 26]. In prior information it has been postulated that there may exist susceptibility genes involved in adipogenesis and energy metabolism [27, 28]; in the current study we explored the distribution of polymorphisms G-2518A and Val64Ile of* CCL2* and* CCR2*, respectively, in individuals with IR.

In addition, the individuals' carriers of different genotypes according to gene dosage were evaluated on the possible association with CCL2 levels, metabolic markers, and adiposity within the population with IR.

In the present work 380 individuals, were classified with or without IR by Stern criteria and obesity by BMI WHO criteria. The frequencies found in IR and obesity, clinical data, and anthropometric measurements are consistent with previous reports in the Mexican population [26, 29].

There are several reasons why a certain obese phenotype possibly will not be equally expressed (e.g., different physical activity levels among participants); however, the aim of the study was to investigate the relationship between the polymorphisms of* CCL2* and* CCR2* with CCL2 soluble levels, metabolic markers, and adiposity (like indicator of body fat status, absolute and/or relative) measured by BIA. Most studies report that the impedance method is reliable and valid. Baumgartner's contribution reviews the assumptions, applicability, equipment, measurement procedure, precision, and accuracy of the BIA method and determined that they were highly recommended [30–32]. Unfortunately we did not evaluate the fat distribution in study subjects; hence, we were not able to include a body fat distribution analysis.

In this set, clinic profile and the ratio of prevalence of IR, presented in the present study, suggest that IR is a complex disease, meaning that different phenotypes are observed with various clinical stages during the development of the pathogenic process. It has been postulated that in course of the natural history of the IR, the hallmark is a low-grade subclinical inflammatory process, in which circulating monocytes infiltrate to adipose tissue, in a redundant manner, and polarize to M1 macrophages, becoming the main producers of chemokines and their receptors [33].

Two important observations lead this study to supporting that adiposity is associated with metabolic markers, in IR development. First, increased adiposity indicators and triglyceride levels were observed, and secondly no differences were found in other components of lipid profile. The first results are in agreement with previous studies [21, 33–35] that was attributed to the presence of obesity; in this case the expansion and accumulation of fat promotes the progress to IR. The second point given by the present study can be explained by the fact that IR is just component during the development of a mayor disease, since dyslipidemia is a later event, that could generate metabolic syndrome [36].

We observe that IR individuals presented higher inflammatory state, due to increased CCL2 levels in contrast to individuals without IR. These results can be explained by the increased levels of expression of adipokines, chemokines, and proinflammatory cytokines associated with a parallel increase in the number, macrophages in the adipose tissue. The later ones, mainly macrophages with M1 phenotype, are crucial in IR, based on the fact that these cells are the important source of proinflammatory markers: TNF-*α*, IL-6, and CRP [2, 33, 37].

Alongside, in the individuals with IR was confirmed the presence of a low-grade inflammatory process represented by the increased levels of sResistin, CRP, and the decreased levels of total adiponectin and the correlation of them with adiposity status, parallel to the increase in BMI, BFR, and metabolic markers.

The importance of the present results lies in the biological relationship that exists between IR and obesity for the development of other diseases, for instance, metabolic syndrome. Insulin has the following functions: on one side, it promotes synthesis of insulin-like growth factor 1, which correlates with fat accumulation, and increase of white adipose tissue; on other side, it is the leader in the deregulations in the secretion of adipokines and metabolic markers that has been associated with certain types of cancer and other chronic diseases such as T2DM [1, 4, 6, 38].

The main pathological mechanism that connect the increase of adipose tissue with IR is the disfunction of the immune system and the establishment of a low-grade subclinical systemic chronic inflammation state, as a result of increased adiposity. This dysregulation of the immune system can be explained on two mechanisms: first, the adipose tissue in obese subjects has an increased amount of infiltrated macrophages who present an M1 phenotype and have an increase in the expression of 4 retinol binding protein (RPB4); and second, it has been found that high concentrations of fatty acids and RPB4 induce the expression of TNF-*α* and the signaling mediated by the toll-like receptor 4 [1, 27].

In this context, the inflammatory process in IR is an underlying clinical sign in the course of the disease and is identified by an increase in levels of inflammatory markers, as well as deregulation in the production of adipokines; in this respect there is evidence based on animal model studies and in humans, in which the important role of white adipose tissue is demonstrated in the maintenance of an inflammatory response associated with the development of IR [6].

There are numerous studies that have found interactions between polymorphisms and development of diseases such as metabolic syndrome. Due to the link of inflammation with development of IR, numerous genes have been studied [2, 28]. The chemokine CCL2 and their CCR2 receptor have been studied in recent years because of their involvement in the recruitment of macrophages to adipose tissue and subsequent differentiation into proinflammatory M1 phenotype [27, 33, 39].

The polymorphism G-2518A, in* CCL2* gene, was identified as a G to A transition; this change is near to a response site of NF-kB and has been speculated that increases affinity to its ligand; this results in increased levels of CCL2 in the bloodstream and further recruitment of macrophages to adipose tissue compared to those individuals exhibiting wild-type allele. Concerning polymorphism in* CCR2*, Val64Ile, is a transition G to A at position 190 of* CCR2*, changing valine codon (GTC) to isoleucine (ATC) at position 64, a conservative change of neutral nonpolar amino acids. This change makes the CCR2A isoform more stable and increases its half-life but does not affect the stability of the isoform CCR2B [1, 40].

On the stage of genetic diversity between populations, the reported frequencies for polymorphic alleles -2518A and 64Ile ranging from 39.1% to 83.2% and 9.5% to 25.6%, respectively, with differences with the frequencies reported in this study (Tables 5(a) and 5(b)), show that wild-type allele changes in some populations, such as Asiatic. In the present study the allele polymorphic frequencies are similar to those reported in Mexican-Mestizos by González-Enríquez et al. and different from Vázquez-Lavista et al. [41, 42].

We found differences when comparing frequencies of alleles of our Mexican population with other populations of different nations, for example, the Asiatic, Caucasian European, or American populations, with a different genetic background, which is explained by the distribution of alleles as result of their anthropological relationships. Since the conquest by the Spanish, European genetics was introduced on the natures, over the centuries. Hence, individuals included in the present study were not pure endogenous Mexican population. Therefore the differences in genotype distribution with other Mexican populations may be because of the fact that in our study group participants were characterized as Mexican-Mestizos of Western Mexico (Tables 5(a) and 5(b)).

In this study group,* CCL2* polymorphism was associated with IR, according to the results showing differences in the distribution of frequencies of genotypes, phenotypes, and alleles, with a higher contribution of the A+ phenotype than A− phenotype for the presence of IR. A+ phenotype was associated with higher levels of total sAdiponectin and lower levels of insulin and HOMA-IR magnitude, while a decrease was shown in BMI and BFR. The opposite was observed for individuals carrying of polymorphic phenotype Ile+; they had an increase in lipid profile; these results show influence of both polymorphisms in the accumulation of body fat and its relation to the metabolic status of individuals.

This can be explained because this is the main tissue involved in the production of adipokines and increases its volume in the presence of a proinflammatory environment [5, 33]. Previous studies have demonstrated the interference of proinflammatory cytokine in the signaling pathway of insulin receptor [27]. Parallel to this stage, we observed increased levels of glucose in individuals with phenotype Ile+ which indicates involvement of this polymorphism in the establishment of IR.

The adiposity status shown in the individuals of this study regarding the polymorphisms in* CCL2* and* CCR2* and its association with metabolic and inflammatory markers, and levels of adipokines have not been previously described for Mexican-Mestizo population.

Among the most important results of this study are that A+ phenotype of* CCL2* and Ile+ of* CCR2* carriers are associated with higher levels of circulating CCL2; this difference is attributed to the fact that this variant generates a more stable form of the receptor CCR2 membrane which has a longer half-life, allowing for longer signaling that in people with wild-type polymorphism variant [40].

Taking all results together, the present work shows new evidence that suggests that genetic factors contribute to the development of IR and this triggers pathobiological processes. These changes could influence the clinical course and severity of obesity-related diseases, such as IR. Therefore, the genetic load on individuals could play an important role in the genesis of diseases observed in obese subjects.

## 5. Conclusions

The most important results in the individuals of the present study are summarized as follows: association of adiposity with metabolic markers alongside with inflammatory state,* CCL2* polymorphism is associated with IR, while* CCR2* was associated with clinical features of IR besides it was seen that the individuals that had both polymorphic phenotypes had increased levels of CCL2.

These data suggests that the* CCL2*A+ phenotype could impact the reduced body fat storage, metabolic and healthy adipokine profile in Mexican-Mestizo individuals. The opposite,* CCR2*64Ile+ phenotype is associated with altered profile of metabolic markers and BMI. All this data suggests that the* CCL2* and* CCR2* polymorphisms and the signaling through this interactions could play a role in the metabolic changes associated with IR in Mexican-Mestizo population.

As a final remark we can conclude that an increase of CCL2 serum levels is associated with the polymorphic phenotypes (A+/Ile+) of the polymorphisms G-2518A in* CCL2* and Val64Ile in* CCR2* in individuals with insulin resistance of Mexican population.

## Figures and Tables

**Table 1:** Anthropometric characteristics and metabolic markers in individuals included in the study.

Measurement	Study group		*P*
	Individuals without IR	Individuals with IR	
*n* (%)	270 (68)	110 (32)	
Age (years)	35 ± 14	34 ± 14	NS
Height (cm)	163.8 ± 5.7	165.7 ± 1.1	NS
Weight (kg)	**68.7 **±** 13.2**	**79.4 **±** 16.3**	**<**0.001^*∗*^
BMI (kg/m^2^)	**24.9 (22.6**–**28.9)**	**28.6 (24.4**–**31.7)**	**<**0.001^+^
Body fat mass (kg)	**20.1 **±** 9.2**	**25.9 **±** 11.3**	**<**0.001^**∗**^
Total body fat mass (%)	**28.8 **±** 9.4**	**32.80 **±** 9.2**	**<**0.001^**∗**^
BFR (kg/m^2^)	**7.03 (5.24**–**9.40)**	**9.40 (6.37**–**12.20)**	0.001^+^
Waist circumference (cm)	**86.5 (77.1**–**93.1)**	**95.0 (83**–**103)**	**<**0.001^+^
Hip circumference (cm)	**99.5 (95**–**105.9)**	**104 (99**–**110)**	0.002^+^
WHR	0.854 (0.68–1.17)	0.866 (0.70–1.28)	NS^+^
WHtR	**0.533 **±** 0.076**	**0.568 **±** 0.085**	**<**0.001^**∗**^
Glucose (mg/dL)	**89 **±** 10**	**98 **±** 18**	**<**0.001^**∗**^
Insulin (*µ*UI/mL)	**8.6 **±** 3.5**	**35.2 **±** 32.5**	**<**0.001^**∗**^
HOMA-IR	**1.86 (1.34**–**2.58)**	**6.05 (4.40**–**9.03)**	**<**0.001^+^
Triglycerides (mg/dL)	**134 **±** 83**	**162 **±** 95**	0.005^**∗**^
Total cholesterol (mg/dL)	184 ± 40	185 ± 34	NS
HDLc (mg/dL)	39.5 ± 15.1	37.6 ± 14.9	NS
LDLc (mg/dL)	111 ± 36	110 ± 31	NS
VLDLc (mg/dL)	**26 **±** 16**	**32 **±** 19 **	0.005^**∗**^
Apo-A1 (mg/dL)	114 ± 25	117 ± 27	NS
Apo-B (mg/dL)	111 ± 32	117 ± 32	NS

*n* = 380. Data are presented as mean ± standard deviation and median (25–75 percentiles). ^*∗*^Student's *t*-test and ^+^Mann-Whitney *U* test with *P* significantly, comparing the groups: individuals with IR versus individuals without IR. IR: insulin resistance; BMI: body mass index; WHR: waist to hip ratio; WHtR: waist to height ratio; BFR: body fat ratio; HOMA-IR: homeostasis model assessment-insulin resistance; HDLc, LDLc, and VLDLc: high, low, and very low density lipoprotein cholesterol, respectively; Apo: apolipoprotein.

**Table 2:** Correlations soluble levels of adipokines and metabolic markers with body adiposity.

Measurements	^*∗*^Weight (kg)	^+^BMI (kg/m^2^)	^+^Body fat mass (%)	^+^BFR (kg/m^2^)	^+^WC (cm)	^+^WHtR
	% Correlation					
sCCL2 (pg/mL)	20.1	29.4	21.8	29.3	25.0	24.8
CRP (mg/L)	30.6	52.7	52.8	53.0	44.8	52.8
sAdiponectin (ng/mL)	−34.7	−25.0	−17.5	−25.1	−23.7	−17.2
sResistin (ng/mL)	43.3	27.1	30.1	27.3	32.5	24.9
Glucose (mg/dL)	22.7	38.1	24.0	35.4	32.5	35.6
Insulin (*µ*UI/mL)	23.1	39.8	29.9	40.1	27.1	26.4
HOMA-IR	25.0	43.6	32.4	43.5	30.5	30.6
Triglycerides (mg/dL)	22.5	34.9	16.9	33.7	40.1	36.0
Total cholesterol (mg/dL)	19.5	32.2	27.3	30.8	37.5	39.6
VLDLc (mg/dL)	22.4	34.2	16.4	33.0	39.6	35.4
Apo-B (mg/dL)	21.0	34.5	17.5	34.3	46.7	44.3

*n* = 380. BMI: body mass index; BFR: body fat ratio; WC: waist circumference; WHtR: waist to height ratio; CCL2: chemokine (C-C motif) ligand 2; HOMA-IR: homeostasis model assessment-insulin resistance; CRP: C reactive protein; VLDLc: very low density lipoprotein cholesterol; Apo: apolipoprotein. Significant differences: *P* < 0.05, ^*∗*^Pearson or ^+^Spearman correlations test.

**Table 3:** Distribution of *CCL2* (G-2518A) and *CCR2* (Val64Ile) gene polymorphism in Mexican-Mestizo population.

	Genotype, *n* (%)			Phenotype, *n* (%)		Allele, *n* (%)	
Study group	*CCL2* G-2518A						
	G/G	G/A	A/A	A+	A−	G	A
Individuals without IR	47 (17.4)	135 (50.0)	88 (32.6)	223 (82.6)	47 (17.4)	229 (42.4)	311 (57.6)
Individuals with IR	32 (29.0)	51 (46.4)	27 (24.6)	78 (70.9)	32 (29.1)	115 (52.3)	105 (47.7)
^*∗*^ *P*	**0.01378**			**0.01131**		**0.01321**	

	Genotype, *n* (%)			Phenotype, *n* (%)		Allele, *n* (%)	
Study group	*CCR2* Val64Ile						
	Val/Val	Val/Ile	Ile/Ile	Ile+	Ile−	Val	Ile

Individuals without IR	173 (64.1)	82 (30.4)	15 (5.5)	97 (35.9)	173 (64.1)	428 (79.3)	112 (20.7)
Individuals with IR	67 (60.9)	37 (33.6)	6 (5.5)	43 (39.1)	67 (60.9)	171 (77.7)	49 (22.3)
^*∗*^ *P*	0.64924			0.94889		0.63925	

*n* = 380. IR: insulin resistance. ^**∗**^Significant differences: Pearson's goodness-of-fit test *χ* ^2^ or Fishers' exact test; G or Val: wild-type alleles; A+ phenotype: A/A plus G/A genotypes; Ile+ phenotype: Ile/Ile plus Ile/Val genotypes.

**Table 4:** Comparisons of body fat measurements and lipid profile between *CCL2* G-2518A and *CCR2* Val64Ile phenotype carriers.

Measurements	*CCL2* G-2518A			*CCR2* Val64Ile		
	Phenotype A+	Phenotype A−	*P*	Phenotype Ile+	Phenotype Ile−	*P*
*n* (%)	301 (79)	79 (21)		140 (37)	240 (63)	
Weight (kg)	72.0 ± 15.55	72.8 ± 13.56	NS	73.2 ± 14.82	71.5 ± 15.32	NS
BMI (kg/m^2^)	**25.9 (22.9–29.1) **	**27.5 (24.3–29.65) **	^+^0.028	**27.3 (23.8–29.7) **	**25.4 (23.0–28.9) **	0.018^+^
Total body fat (%)	29.6 ± 9.81	31.7 ± 8.57	NS	31.2 ± 9.05	29.4 ± 9.85	NS
BFR (kg/m^2^)	**7.31 (5.24–9.92) **	**8.25 (6.24–11.40) **	^+^0.039	7.63 (5.57–10.90)	7.37 (5.31–9.93)	NS
Waist circumference (cm)	88.0 (78.4–98.0)	89.4 (79.3–96.5)	NS	89.1 (79.5–99.0)	88.1 (78.3–97.6)	NS
Glucose (mg/dL)	92 ± 12	93 ± 14	NS	**95 ± 13**	**91 ± 12**	0.004^**∗**^
Triglycerides (mg/dL)	142 ± 85.6	146 ± 94.2	NS	**156 ± 8.5**	**135 ± 5.0**	0.039^**∗**^
Total cholesterol (mg/dL)	181 ± 40.1	185 ± 34.3	NS	**190 ± 43.7**	**180 ± 35.5**	0.031^**∗**^
HDLc (mg/dL)	39 ± 14.8	37 ± 15.8	NS	38 ± 13.6	38 ± 15.8	NS
LDLc (mg/dL)	111 ± 35.6	109 ± 30.4	NS	114 ± 39.1	108 ± 31.2	NS
VLDLc (mg/dL)	28 ± 16.9	29 ± 18.8	NS	**31 ± 20.1**	**26 ± 15.3**	0.032^**∗**^
Apo-A1 (mg/dL)	114 ± 25.1	121 ± 29.1	NS	117 ± 24.4	114 ± 27.1	NS
Apo-B (mg/dL)	112 ± 30.0	121 ± 41.5	NS	**119 ± 36.8**	**109 ± 27.8**	0.045^**∗**^

*n* = 380. BMI: body mass index; BFR: body fat ratio; HDLc, LDLc, and VLDLc: high, low, and very low density lipoprotein cholesterol, respectively; Apo: apolipoprotein. A− or Ile−: wild-type phenotypes; polymorphic A+ phenotype (A/A plus G/A genotypes); polymorphic Ile+ phenotype (Ile/Ile plus Val/Ile genotypes). Data are presented as mean ± standard deviation and median (25–75 percentiles). ^*∗*^Student's *t*-test and ^+^Mann-Whitney *U* test with *P* significantly, comparing the polymorphic phenotype carriers versus wild-type phenotypes carriers.

**(a):** 

Author	Population	*n*	G	A	G/G	A/G	A/A	*P*
The present study	**Mexican-Mestizo**	**380**	**45.2**	**54.6**	**20.7**	**48.9**	**30.3**	—
Vázquez-Lavista et al. [41]	Mexican-Mestizo	126	57.5	42.5	29.4	56.3	14.3	**<0.001**
González-Enríquez et al. [42]	Mexican-Mestizo	21	52.4	47.6	27.0	50.0	23.0	0.370
Bektas-Kayhan et al. [24]	Caucasian	140	16.8	83.2	0.8	32.1	67.1	**<0.001**
Kruszyna et al. [43]	Caucasian	323	29.9	70.1	7.4	44.9	47.7	**<0.001**
Kucukgergin et al. [44]	Caucasian	197	30.2	69.8	9.1	42.1	48.7	**<0.001**
Kouyama et al. [13]	Asian	361	34.6	65.4	—	—	—	—
Singh et al. [7]	Asian	200	35.3	64.7	11.0	48.5	40.5	**<0.001**
Mandal et al. [45]	Asian	390	37.2	62.8	13.2	48.0	38.8	**0.001**
Chen et al. [23]	Asian	344	51.7	48.3	26.7	50.0	23.3	**0.015**
Wu et al. [46]	Asian	253	60.1	39.1	34.8	52.2	13.0	**<0.001**

Pearson's goodness-of-fit test *χ* ^2^ or Fishers' exact test with *P* significantly, comparing alleles and/or genotypes polymorphism distribution in Mexican-Mestizos population in this study versus distribution in other populations. G wild-type allele, A polymorphic allele.

**(b):** 

Author	Population	*n*	Val	Ile	Val/Val	Val/Ile	Ile/Ile	*P*
The present study	**Mexican-Mestizo**	**380**	**78.8**	**21.2**	**63.2**	**31.3**	**5.5**	—
Vázquez-Lavista et al. [41]	Mexican-Mestizo	126	75.8	24.2	58.7	34.1	7.2	0.330
González-Enríquez et al. [42]	Mexican-Mestizo	21	90.5	9.5	82.6	17.3	0	**0.006**
Bektas-Kayhan et al. [24]	Caucasians	140	88.5	11.42	80.0	17.1	2.9	**<0.001**
Kucukgergin et al. [44]	Caucasians	197	89.6	10.4	80.7	17.8	1.5	**<0.001**
González et al. [47]	Caucasians	280	90.0	10.0	80.0	19.0	1.0	**<0.001**
Mandal et al. [45]	Asian	390	74.4	25.6	54.8	39.2	6.0	**0.046**
Wu et al. [46]	Asian	253	80.6	19.4	64.4	32.4	3.2	0.438
Singh et al. [7]	Asian	200	80.5	19.5	63.0	35.0	2.0	0.501
Zandifar et al. [48]	Asian	100	87.5	12.5	75.0	25.0	0	**0.007**
Chen et al. [23]	Asian	344	89.1	10.9	80.2	17.7	2.1	**<0.001**

Pearson's goodness-of-fit test *χ* ^2^ or Fishers' exact test with *P* significantly, comparing alleles and/or genotypes polymorphism distribution in Mexican-Mestizos population in this study versus distribution in other populations. Val: wild-type allele; Ile: polymorphic allele.
